# Developing integrated crop knowledge networks to advance candidate gene discovery

**DOI:** 10.1016/j.atg.2016.10.003

**Published:** 2016-11-02

**Authors:** Keywan Hassani-Pak, Martin Castellote, Maria Esch, Matthew Hindle, Artem Lysenko, Jan Taubert, Christopher Rawlings

**Affiliations:** a Rothamsted Research, Department of Computational and Systems Biology, UK; b INTA EEA-Balcarce, Laboratory of Agrobiotechnology, Argentina

**Keywords:** GSKN, Genome-scale Knowledge Network, CropNet, Crop knowledge network, RefNet, Reference knowledge network of model species, Bioinformatics, Knowledge network, Data integration, Gene discovery, Knowledge discovery, crop genomics

## Abstract

The chances of raising crop productivity to enhance global food security would be greatly improved if we had a complete understanding of all the biological mechanisms that underpinned traits such as crop yield, disease resistance or nutrient and water use efficiency. With more crop genomes emerging all the time, we are nearer having the basic information, at the gene-level, to begin assembling crop gene catalogues and using data from other plant species to understand how the genes function and how their interactions govern crop development and physiology. Unfortunately, the task of creating such a complete knowledge base of gene functions, interaction networks and trait biology is technically challenging because the relevant data are dispersed in myriad databases in a variety of data formats with variable quality and coverage. In this paper we present a general approach for building genome-scale knowledge networks that provide a unified representation of heterogeneous but interconnected datasets to enable effective knowledge mining and gene discovery. We describe the datasets and outline the methods, workflows and tools that we have developed for creating and visualising these networks for the major crop species, wheat and barley. We present the global characteristics of such knowledge networks and with an example linking a seed size phenotype to a barley WRKY transcription factor orthologous to TTG2 from Arabidopsis, we illustrate the value of integrated data in biological knowledge discovery. The software we have developed (www.ondex.org) and the knowledge resources (http://knetminer.rothamsted.ac.uk) we have created are all open-source and provide a first step towards systematic and evidence-based gene discovery in order to facilitate crop improvement.

## 1 Introduction

The development of improved agricultural crops is a critical societal challenge, given current global developments such as population growth, climate and environmental change, and the increasing scarcity of inputs (fuel, fertiliser, etc.) needed for agricultural productivity. To meet this challenge, we will need to breed or engineer improved crop varieties, with higher yields, robustness to biotic (e.g. pathogens, pests) and abiotic stress, and better adapted to their intended environments; and to accelerate the breeding programmes needed to implement these designs. Many biomedical and agronomic traits are complex and their variation is determined by manifold interactions among genetic, epigenetic and environmental factors (Polderman et al., 2015). The use of forward genetics, reverse genetics and “omics” technologies combined with bioinformatics approaches to discover causal genetic loci and variants that determine a particular biological phenotype in crops, animals or humans is referred to as the genotype to phenotype challenge.

The generation of the hypotheses that link genotype to phenotype and the identification of the candidate biological pathways, processes and functional genes that could be involved requires the integration of multiple heterogeneous types of information. This information is spread across many different databases (Rigden et al., 2016) that can include known records of gene-phenotype links, gene-disease associations, gene expression and co-expression, allelic information and effects of genetic variation, links to scientific literature, homology relations, protein-protein interactions, gene regulation, protein pathway memberships, gene-ontology annotations, protein-domain information and other domain specific information. Such data is typically highly connected, e.g. through common references to named biological entities, and semi-structured, e.g. because some data can be found in databases and other in free text. Furthermore, these data types are not static because new types of data are constantly emerging from advances in high-throughput experimental platforms. These characteristics of life science data make networks, consisting of nodes and links between them, represent a flexible data model that can capture some of the complexity and interconnectedness in the data (Huber et al., 2007).

In mathematics and computer science a distinction is made between homogeneous networks, where all nodes have the same data type (e.g. protein-protein interaction networks) and heterogeneous networks, sometimes referred to as information or knowledge networks, where nodes and links can have various types (Sun and Han, 2012). Biological knowledge networks are composed of nodes which represent biological entities such as genes, transcripts, proteins and compounds, as well as, other entities such as protein domains, ontology terms, pathways, literature and phenotypes (Liekens et al., 2011). The links in the network correspond to relations between entities and are described using terms which reflect the semantics of the biological or functional relationship such as *encodes*, *interacts*, *controls*, *expressed*, *part_of, is_a*, *published_in*, etc. A knowledge network is referred to as genome-scale knowledge network (GSKN) when it contains the entire known genome (all genes) of an organism as nodes in the network. An integrated GSKN built from a semantically rich variety of dispersed, heterogeneous data can add value to the native data and significantly facilitate both computer-aided data mining and manual data exploration.

There are a variety of ways to represent information in knowledge networks. Simple information such as a gene location can be added as start and end attributes (key-value-pairs) on the nodes representing genes (Gene nodes). However, when the nature of the information is more complex, it can be better represented in an expanded form with additional linked nodes of clearly defined types. For example, SNP information could either be represented in a simplistic manner, i.e. as multiple attributes on a Gene node or in a linked way using individual Gene-SNP relations. The linked approach enables nodes representing SNPs (SNP nodes) to have properties attached to them and allows further linking of SNP-Phenotype information, for example, based on the results of a genome wide association study (GWAS). Therefore, representing this information in an expanded form with clearly defined types can enable better data visualisation and more effective data mining.

**Fig. 1:**
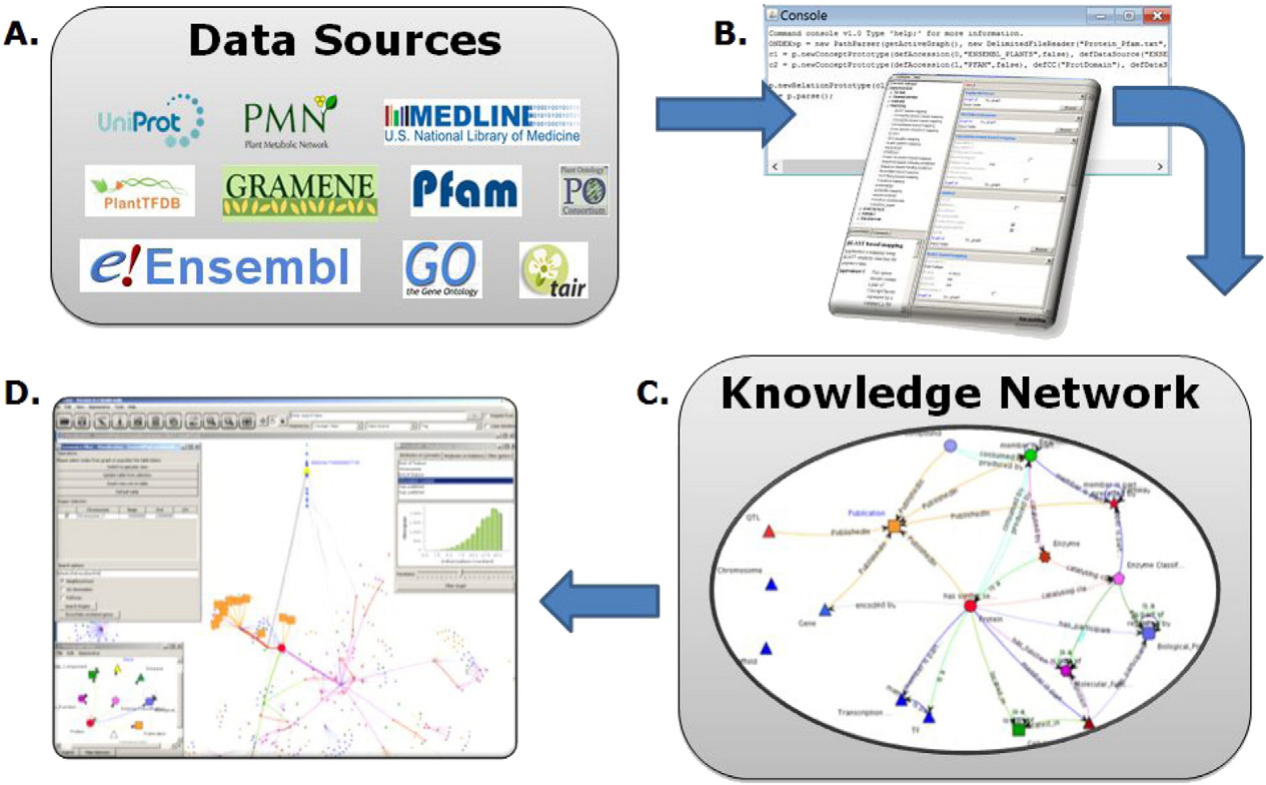
Examples of public data sources that can be integrated into Ondex (A) using the Ondex Integrator and the Ondex Console (B). Following the data integration workflow, the knowledge network (C) is loaded into the Ondex UI for visualisation and exploration (D).

Ondex provides a framework for building integrated knowledge networks from heterogeneous datasets (Köhler et al., 2006). In Ondex terminology, the nodes of a network are called concepts and the links between them are called relations. To address a certain integration or analysis task in Ondex, public and private data sources containing the desired type of information need to be selected. The Ondex framework uses a network data model and provides an Application Program Interface (API) to insert data into the data model. The Ondex in-memory network data structure is based on a labelled and directed multi-graph that is flexible and allows information and metadata from most biological databases to be captured. Ondex uses a set of parsers implemented as plugin software modules that transform data from their native format and exploit the Ondex API to populate the network. Other plugin modules perform data integration tasks that identify semantically similar nodes (mapping methods), remove unwanted information (filters) and simplify the network structure (transformers). These plugins can be combined together using an XML-based description of a workflow of reproducible steps that convert heterogeneous data into integrated networks. Such Ondex workflows can be generated and executed either via a graphical user interface (Ondex Integrator) or via the command line interface (Ondex-CLI). The Ondex Scripting Console provides a means to parse configurable tabular data formats (including simple CSV format files) for integration into Ondex where no format-specific Ondex parsers are yet available. The scripting syntax is based on a bespoke language developed in Lysenko 2012. Ondex networks can be exported in several formats such as the Ondex exchange format OXL (Taubert et al., 2007), RDF (Splendiani et al., 2012) or Cytoscape-compatible JSON. Networks can be visualised and inspected using tools like Cytoscape (Shannon et al., 2003) or in the Ondex User Interface (Ondex UI) (Fig. 1).

Since its release, Ondex has undergone various phases of development. Recent work has extended the Ondex UI with an on-demand information retrieval capability using web-service based scripts that add the retrieved information to a visualised network (Horn et al., 2014). This enables an exploratory analysis: starting with a small network and then gradually, on-demand, extending to a larger network. A web-enabled version of the Ondex UI, called Ondex Web, has been developed to allow Ondex network visualisations to be embedded in web-pages (Taubert et al., 2014). Furthermore, a Cytoscape plugin, called OndexView allows for concise graphical representations of integrated knowledge networks (Weile et al., 2011). Some studies were undertaken to evaluate the utility of semantic web technologies (RDF, SPARQL) within the umbrella of Ondex (Splendiani et al., 2012, Canevet et al., 2010). Two studies showed the contributions of Ondex towards Bayesian data integration (Weile et al., 2012) and towards logic-based modelling (Lesk et al., 2011). Finally, Ondex was used as the main platform for biological network analysis (Lysenko et al., 2011, Defoin-Platel et al., 2011) and in a biological study to identify candidate virulence genes in the fungus *Fusarium graminearum* (Lysenko et al., 2013).

## 2 Methods and principles

The creation of GSKNs for crop species is described in the following four sections: i) the general principles of data integration in Ondex, ii) building a reference knowledge network from model species datasets (RefNet), iii) integrating crop-specific information (CropNet) and iv) the steps needed to update the knowledge networks.

### 2.1 Ondex approach to data integration

**Fig. 2:**
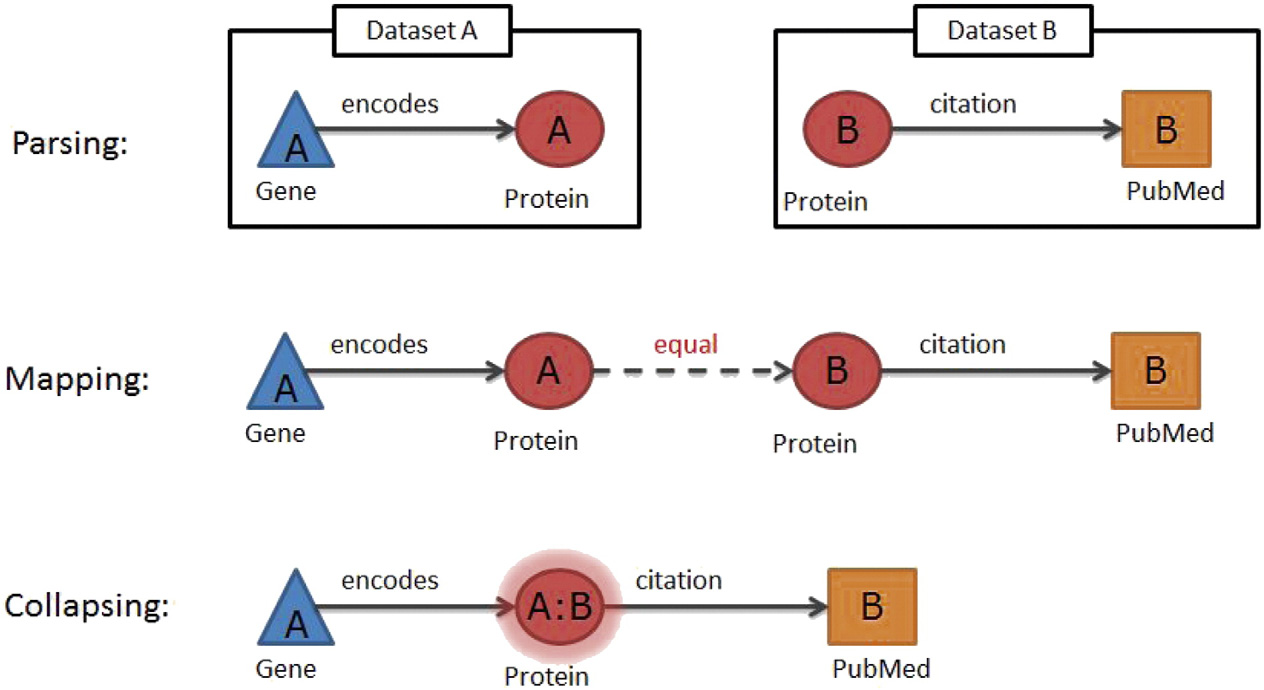
The Ondex workflow involves parsing, mapping and collapsing the data. Ondex input datasets A and B are merged via common concepts (e.g. Protein). The mapping step creates relations of type *equal* between “equivalent” concepts. The collapsing is a network transformation that merges equivalent concepts into a single concept to avoid redundancy. The merged concepts contain a summary of all the data sources as a record of the provenance of the merged network.

We illustrate the Ondex approach to knowledge network creation by integrating a small knowledge network from three different data sources UniProt (UniProt Consortium, 2015), Gene Ontology (GO) (Gene Ontology Consortium, 2015) and PubMed (http://www.ncbi.nlm.nih.gov/pubmed). The goal is to merge these different data sources in order to gain insights from studying their intersection. The main steps towards achieving this goal include parsing data, mapping equivalent concepts and collapsing redundant concepts (Fig. 2).

#### 2.1.1 Parsing

Ondex uses an ontology model that describes what and how data are captured in an Ondex network. This ontology is the core semantic framework for the data model and describes Ondex *Concept Classes*, *Relation Types*, *Data Sources*, *Attribute Names* and *Evidence Types*. Every external data source is parsed so that it becomes a network populated with instances from the ontology model. Parsers are plugins in Ondex that read each dataset and produce independent networks within Ondex. Concepts and relations of an Ondex network will capture the parsed content within their attributes. An alternative way of importing data into Ondex is through the Ondex Scripting Console. Ondex scripts can for example parse a tabular file that contains gene IDs in one column and gene-related SNP data in the other columns. The code can create *Gene* and *SNP* concepts for every line in the tabular file and a relation of type *has_variance* that connects the two concepts. Concepts sharing the same accessions within the parsed dataset are automatically merged into one representative concept.

#### 2.1.2 Mapping

Importing UniProt, GO and PubMed into Ondex creates individual Ondex networks for each dataset. Each of these networks may have information overlapping with the other networks that may provide additional information about particular concepts or relations. For example, UniProt has references to GO and PubMed, but lacks the hierarchy of GO and the abstracts from publications that are necessary for proper querying and analysis. This missing information is contained within the Gene Ontology OBO and PubMed XML files. Thus, the GO concepts in the UniProt network need to be mapped to corresponding concepts in the Gene Ontology network. This process of mapping in Ondex enables a flexible and pragmatic approach to identifying equivalent concepts between databases. For instance, the Ondex “*accession based mapping*” can be used to create a relation of type *equal* between two concepts of the same type when they share a common unique identifier such as GO or PubMed IDs. Alternative mapping approaches can be used for mapping concepts with no common identifiers. These include “*name based mapping*” that maps based on shared names / synonyms or “*sequence based mapping*” that maps based on the similarity of sequence attributes.

#### 2.1.3 Collapsing

After mapping related concepts using exactly matching common database accessions, all equivalent concepts can be collapsed into a single concept. The mapping and collapsing steps ensure that no concept occurs more than once in the network. Avoiding redundancy in the construction of the knowledge network makes successive data mining of the integrated resources significantly easier. In Ondex, this can be done with a network transformer such as the “*Relation collapser*”. The provenance of the data is stored within the *Data Source* attribute of each Ondex concept. Once two or more concepts have been collapsed, this attribute will be assigned a summary of all the data provenances. This is how Ondex keeps track of the source of the data. These mentioned steps interconnect several data sources into one integrated knowledge network.

### 2.2 Integration of model species data

The majority of genes in crop species such as wheat or barley are not well studied and have vague or unknown functions. Therefore, to be able to link the genetic and genomic crop information to biological processes and pathways we need to infer functional gene information from model species based on sequence conservation and comparative genomics. For most plant and crop species, the most suitable model species, with a range of high quality annotation and interaction data is *Arabidopsis* (Defoin-Platel et al., 2011). We also like to include other well-studied plant species that provide high quality functional gene information. From these model species datasets, we follow an integration approach to build a reference knowledge network (a RefNet) that can be added to crop-specific networks.

Curated Arabidopsis and plant datasets can be retrieved from public databases such as TAIR (Lamesch et al., 2012), Araport (Krishnakumar et al., 2015), Gramene (Jaiswal, 2011) and UniProt. Using the Ondex Integrator and similar integration principles (parsing, mapping and collapsing) as before, a core Arabidopsis network was first developed consisting of *Gene-Protein* relations. This core network can be iteratively extended with functional and interaction data including GO annotations, Gramene Trait Ontology (TO), pathway data, phenotypes, GWAS data, protein-protein interactions (PPI) and links to relevant publications. The RefNet was created by interconnecting these individual datasets based on mapping and collapsing equivalent *Gene* or *Protein* concepts. The PPI dataset provided by TAIR is based on Arabidopsis gene identifiers and not protein ids, therefore, it was translated into a *Gene-Gene* interaction network. Next, all reviewed plant proteins (excluding Arabidopsis) with their GO annotations and literature citations were retrieved from UniProt and added to the RefNet.

Gene-phenotype information based on experimental evidence are the most valuable pieces of evidence in trait-based gene discovery. Such connected phenotype information is available in dispersed locations including UniProt, TAIR and NCBI GeneRIF databases. The majority of plant phenotype descriptions, however, is still only available as unstructured text without a mapping to a structured phenotype ontology. In the Arabidopsis knowledge network, gene-phenotype information curated by domain specialists is represented as *Phenotype* concepts linked to genes or as attributes of protein concepts from UniProt. A certain amount of redundancy can occur when the data providers have not used ontologies to describe same/similar phenotypes. In addition to curated gene-phenotype relations, automated text-mining methods can be exploited to extract and integrate phenotypic information from PubMed abstracts and link them to the corresponding Arabidopsis genes. The Trait Ontology (TO) and Arabidopsis gene names were used as inputs to the Ondex text-mining plugin (Hassani-Pak et al., 2010) which established new relations between *Gene* and *TO* concepts related to plant developmental and functional traits. Additional filtering steps in the RefNet workflow helped to retain only those relations where gene names and ontology terms co-occurred in the same sentence. Tn total, over 27,000 new text-mining based *Gene-TO* relations were added to the knowledge network.

### 2.3 Integration of crop specific data

The methods and workflows to build crop-specific knowledge networks (CropNet) are presented with data from barley and wheat but are similarly applicable to other crop species.

#### 2.3.1 Genes and proteins

The starting point of building a genome-scale knowledge network is a public genome sequence dataset. Our interest is not in the genome sequence itself but more in the annotations including genes and the transcripts/proteins they encode. These annotations are available from GFF3 files (The Sequence Ontology - Resources - GFF3 [Internet]) and sequence information can be obtained in FASTA format. The Ondex “FASTA-GFF3” parser takes standard GFF3 and protein FASTA files as inputs and produces a network of *Gene* and *Protein* concepts connected via relations of type *encodes*. Information such as chromosome, start and end are added as attributes of the Gene concepts. A taxonomy identification argument (TAXID) is used to distinguish which species (e.g. wheat) the data comes from. Wheat and barley gene models and protein sequences were obtained from Ensembl and parsed using the FASTA-GFF3 Ondex parser. This creates the *Gene-Protein* network in which concepts are connected via relations of type *encodes*.

The “TSV file parser” can be used to add simple information such as gene location or synonymous names to the Gene concepts. The parser creates a concept for every line in a tabular file and adds information from the columns as attributes of the concept. This information can be mapped and collapsed and provides a useful way for adding missing attributes to concepts in the knowledge network. The wheat POPSEQ dataset provides estimated gene locations in centiMorgans (cM) based on a whole genome sequencing approach (Chapman et al., 2015). The “TSV file parser” was used to add the POPSEQ-based cM coordinates to the wheat *Gene* concepts.

#### 2.3.2 Genetics and genome variation

The next integration goal is to incorporate genome variation (i.e. SNPs) and genetics data (i.e. GWAS and QTL) into the knowledge networks. In order to ensure that GWAS and QTL data can be co-located with genes it is important that the datasets are based on the same physical or genetic maps. In the ideal case, every gene in the network will have a chromosome, start and stop position based on physical genome coordinates (bp), and similarly SNP and QTL intervals would be defined using genome location. For many crop species, QTLs are defined by their genetic map locations. In such cases, QTL intervals need to be transformed from genetic to physical map coordinates before being incorporated into the knowledge network. An ideal resource for standardised QTL and GWAS data of livestock species in GFF3 format is the AnimalQTLdb (Hu et al., 2016). In crop species, however, such structured genetics resources are only slowly beginning to emerge (Blake et al., 2016). Once such data is in the public domain, the Ensembl Variation database provides access to the variation data, along with genes in close proximity, functional consequence of the variation and associations with phenotypes (Chen et al., 2010). The Ondex Scripting Console can be used to translate such tabular data into *Gene-SNP-Trait* and *QTL-Trait* networks. The *Gene-SNP-Trait* network was mapped to the *Gene-Protein* network based on common ENSEMBL gene ids (e.g. TRAES_2AL_65B19CC73). The *QTL-Trait* network was integrated with the *Gene-Protein* network by using a mapping method that creates links between *Gene* and *QTL* concepts when they are co-located.

#### 2.3.3 Orthology and protein domains

The next step in building CropNet is to enrich and extend the wheat *Gene-Protein* network with novel links based on comparative sequence analysis, which includes orthology, conserved protein domains and sequence similarity to protein databases. Such information may sometimes be available as pre-computed datasets for download from public databases such as Ensembl (Herrero et al., 2016), Phytozome (Goodstein et al., 2011) or OMA Browser (Altenhoff et al., 2014). It can also be locally computed using tools like OMA Standalone, InterProScan (Mitchell et al., 2015), Smith-Waterman (SW) (Smith and Waterman, 1981) or Blast (Altschul et al., 1990). It is important that the *Protein* concepts in the RefNet have the same accessions as provided in the orthology and sequence similarity based datasets of CropNet, since these will be used to interconnect the two networks. Pre-computed data from wheat included protein domains and inferred orthology to *Arabidopsis* and barley were obtained from Ensembl BioMart. Sequence alignments between all wheat proteins and reviewed UniProt plant proteins (excluding *Arabidopsis*) were created using SW while taking the top 10 hits per query sequence (*E*-value < 0.001). The Ondex Scripting Console was used to parse these additional wheat related datasets, transform them into Ondex networks and export them in OXL format. These steps created new concepts of type *Protein* and *Protein Domain* and new relations of type *ortholog, has_domain* and *has_similar_sequence* that were integrated with the *Protein* concepts of wheat *Gene-Protein* network based on ENSEMBL protein ids (e.g. TRAES_2AL_65B19CC73.1).

#### 2.3.4 Integrating CropNet with RefNet

The above steps were followed to create a knowledge network for the crop of interest (a CropNet), which contains genes, proteins, genetic, orthology and protein domain information. In a final integration step, the CropNet and the RefNet were brought together and linked using the orthology and protein domain information. Their integration was based on common concept accessions so that duplicated concepts were mapped and collapsed. For example, CropNet contains both wheat *Protein* concepts and *Protein* concepts from the reference species. These functioned as anchors for linking the two networks through accession-based mapping steps by mapping and collapsing *Protein* concepts using shared TAIR and UniProt accessions. Additionally, *Protein Domain* concepts from the CropNet were connected to corresponding GO terms in the RefNet. This step exploited public GO mapping files (Index of /external2go [Internet]) as the input to the Ondex mapping plugin *External2GO*. This mapping method created relations of type *“cross_reference”* between semantically similar concepts of different type. A full overview of selected data sources and Ondex parsers used for building a crop GSKN are given in Table 1.

### 2.4 Updating knowledge networks

Public life science databases are not static and are updated regularly. For example, the number of publications in PubMed that contain the word Arabidopsis has risen by nearly 20,000 new articles in the last 5 years. In recent times, GO annotations, nucleotide and protein sequence repositories have all had a similar sharp rise in their content. Therefore, it is increasingly important to keep such fast growing information up-to-date on a regular basis. On the other hand, crop-specific datasets such as genome assemblies, gene models and QTL data change less frequently and can therefore be updated on demand.

In order to facilitate the continuous reintegration of updated data we created a central knowledge store of data snapshots that correspond to the database versions available at the time of integration. We have developed scripts for rebuilding this knowledge store in a semi-automated manner. The focus has been on automating the update of datasets that frequently change and are publicly accessible in standardised formats for which Ondex Parsers are available, i.e. ontologies, publications and GO annotations. We have therefore automated most of the RefNet data download and integration steps that include:

● Backup of old RefNet datasets
● Download and integrate new RefNet datasets including
  ○ UniProt plants (XML format)
  ○ Gene ontology (OBO format)
  ○ Trait ontology (OBO format)
  ○ Arabidopsis Gene Annotations (GAF)
  ○ PubMed abstracts (XML format)
  ○ BioGRID interactions (TAB)
● Re-run workflows that integrate new RefNet with existing CropNet
● Export new GSKN in OXL or other formats

The update and integration scripts make use of Ondex-CLI which is a lightweight version of Ondex that runs on the command line and not via a graphical user interface. The results are manually inspected in the Ondex UI and by studying the integration logs.

## 3 Results

### 3.1 Comparison of crop knowledge networks

We have developed reproducible workflows to integrate multiple public data sources from crop and model species into genome-scale knowledge networks (GSKN). The Ondex integration process lasts ~ 5 h on a Linux server with Intel E7 2.2 GHz and requires 20 Gb RAM to build a GSKN for species like wheat and barley. All workflows and datasets for building crop GSKNs are available online, e.g. see the release notes of the wheat GSKN (KnetMiner Wheat Release Notes [Internet], 2016).

Each crop GSKN includes an identical reference network consisting of *Arabidopsis* and other plant species from UniProt-SwissProt Plants but differs in their crop-specific information (genes, SNP, QTL, traits, publications). The size of a crop GSKN can vary depending on the genome size and data integrated for that particular organism. The wheat and barley genome releases that were integrated contained 99,386 and 79,379 genes respectively. The resulting version of the wheat GSKN (WheatNet) contains about 450 k concepts and 1.7 million relations, while the barley GSKN (BarleyNet) is slightly smaller with 420 k concepts and 1.3 million relations. The type and amount of information held in a knowledge network also varies from species to species. Tables 2 provides a comparison of the abundance of *Concept Classes* in BarleyNet and WheatNet. In barley, we integrated trait, QTL and SNP information from Gramene and Ensembl resulting in 30 quantitative traits, 285 QTL and 16,030 SNP concepts in BarleyNet. At the time of this analysis, such data was not readily available for wheat in standardised formats but can be added to WheatNet once it becomes available. In both wheat and barley GSKNs, nearly 10% of genes consist of only a simple *Gene-Protein* network; these are genes for which the only available information are the proteins they encode (without genetic, homology or protein domain data). The text-mining analysis connected 5553 *Arabidopsis* genes to 409 TO terms based on 18,341 co-citations.

### 3.2 Search and visualisation of GSKN in Ondex

Due to the scale of GSKNs, visualisation and interaction with such large and complex networks is not straightforward. Opening a GSKN in the Ondex visualisation application takes about 5 min (Windows PC with Intel Core i5 CPU) and requires at least 6 Gb of RAM for storing the network in-memory.

**Fig. 3:**
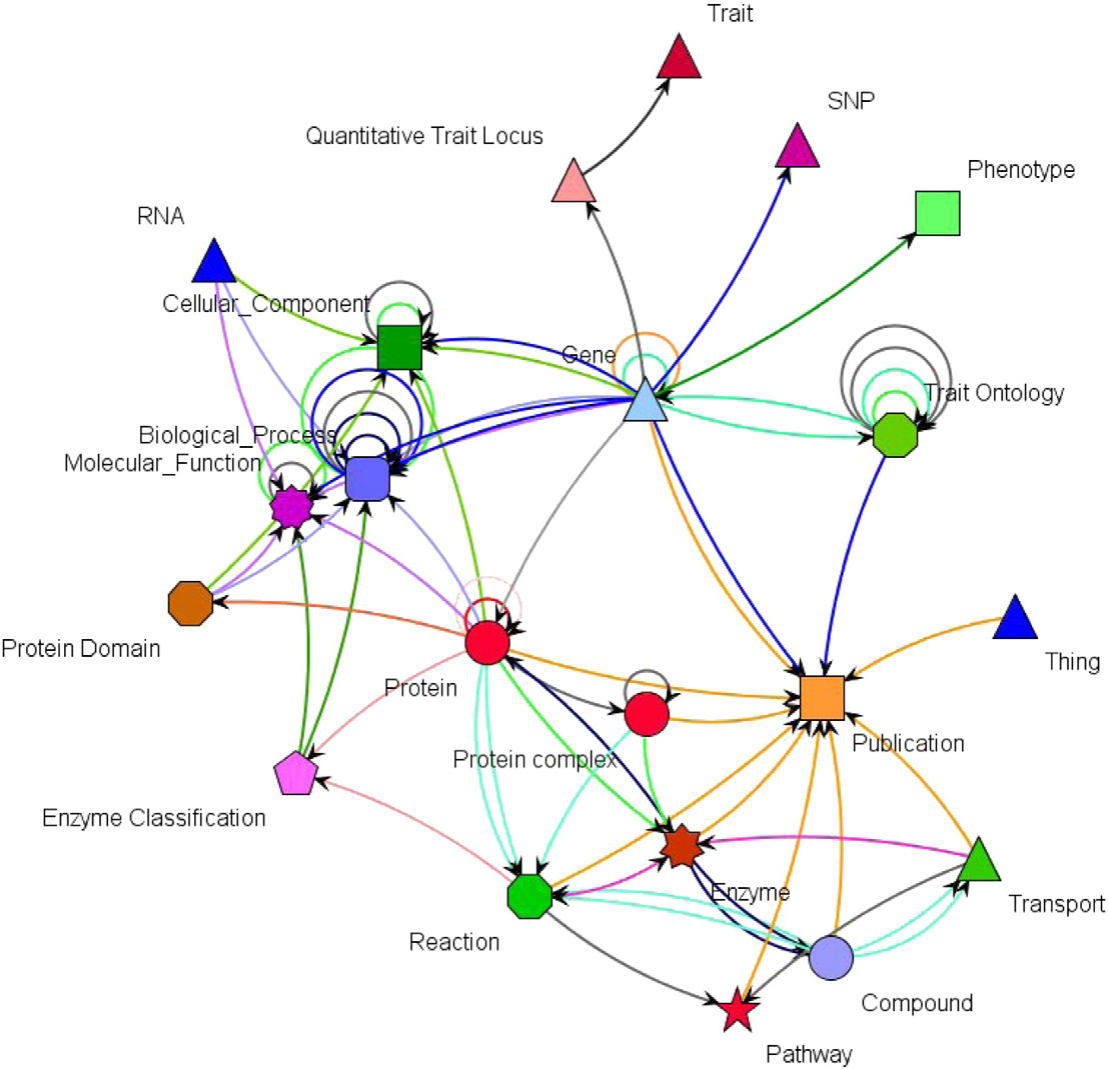
The ontology types present in the metagraph of the barley GSKN (BarleyNet). Different node shapes and colors represent different Concept Classes. Relation Types are omitted here for clarity reasons.

Although the main network is too large to be displayed, the ontology types, represented in the Ondex Metagraph visualisation, summarise the various *Concept Classes* and *Relation Types* present in the knowledge network and their relationships. Fig. 3 shows the ontology types present in the metagraph of the barley GSKN. The metagraph consists of 21 different *Concept Classes* representative of both biological entities (Gene, Protein, Protein Complex, Compound, SNP) and general entities (Biological Process, Pathway, Phenotype, Publication). The metagraph visualises relationships between *Concept Classes*, for example, ‘Biological Process (GO)’ has incoming relations from multiple *Concept Classes*, such as Gene, Protein, RNA, Protein Domain and Enzyme Classification. The Metagraph functions enable users to make subsets of the main network visible/invisible.

In addition, Ondex provides graphical user interfaces for searching, filtering and annotating networks that can be used to gradually build small to medium size networks for visualisation. For example, it is possible to search for genes or phenotypes of interest and apply a neighbourhood search or a shortest path search to identify a potential link between selected concepts. Such smaller subnetworks can be gradually extended using the context-sensitive right-click menus in Ondex that allow additional links of certain types to be added to the knowledge network (Horn et al., 2014).

### 3.3 Application of GSKN to gene discovery and crop improvement

**Fig. 4:**
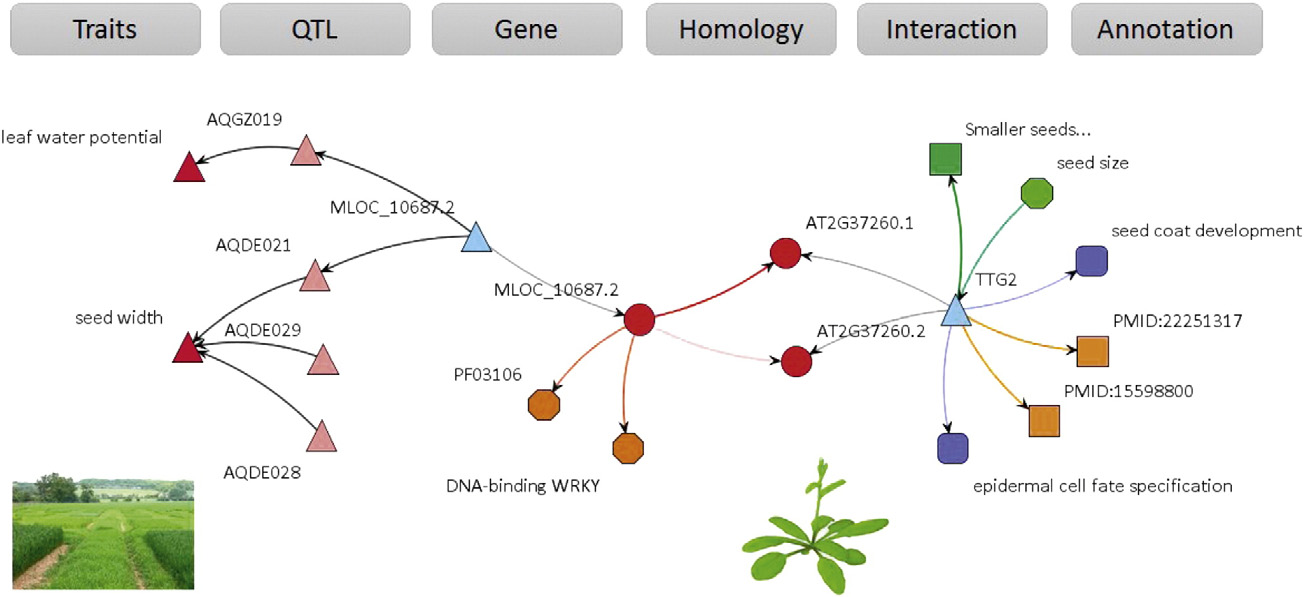
A heterogeneous knowledge network that links crop-specific information on the left (Traits, QTL and Gene) to RefNet information on the right (Homology, Interaction and Annotation).

The main driver for building crop GSKNs was that selected data sources contain fundamental relationships that upon integration and consecutive analysis, can yield chains of functional associations among more distant concepts. In this case, the motivation was to identify chains of functional associations between traits (phenotypes) and causal genes. Here, we present an example network, extracted from the BarleyNet, to provide a proof-of-concept and demonstrate the potential application of integrated data to biological knowledge discovery. The BarleyNet was searched and filtered to identify a potential relationship between barley gene MLOC_10687.2 and a seed size phenotype (Fig. 4). The analysis extracted a gene-evidence network that shows MLOC_10687.2 to be co-located with QTLs on chromosome 5 for seed width (AQDE021, http://archive.gramene.org/db/qtl/qtl_display?qtl_accession_id=AQDE021) and leaf water potential (AQGZ019). It encodes a protein that contains a DNA-binding WRKY domain and is orthologous to *TTG2* in Arabidopsis. Evidence information in Arabidopsis indicates that *TTG2* mutants have smaller seeds and that *TTG2* is involved in seed coat development and epidermal cell fate specification. PubMed references (PMID:22251317 and PMID:15598800) are provided within the knowledge network as an additional source of evidence (Fang et al., 2012, Garcia et al., 2005).

This example highlights the potential benefits of data integration and linked data to establish associations between distant concepts such as traits/QTL on the one side and genes/biological processes on the other side. The original information was dispersed across several heterogeneous databases (Gramene, Ensembl, TAIR, GO and PubMed) and only by interconnecting them in a semantically consistent manner it is possible to search the information effectively and reveal indirect transitive associations between distant concepts of the network. This and other similar examples provide a proof-of-concept for data integration needs in life sciences.

## 4 Discussion

To demonstrate a general approach for building genome-scale knowledge networks (GSKNs) we have presented, as examples, those we have developed for the cereal crops wheat and barley. These GSKNs can be used for the identification of candidate genes and the generation of research hypotheses. The flexibility of the network construction approach is readily transferable to other plant and animal species. We have, in fact, developed knowledge networks for several other species such as *Arabidopsis*, poplar, potato, tomato, *Brassica*, maize, pig, cattle and chicken. Resulting networks are provided in different formats that can be explored in tools like Ondex frontend or Cytoscape. Due to the large size of GSKNs, the exploration and visualisation of such networks is resource intensive, slow and requires powerful computers with sufficient RAM. Tools for knowledge mining and discovery need to be further developed to exploit integrated knowledge networks more effectively in order to predict candidate genes for key agronomic traits in a systematic manner. We are currently in the process of developing such a tool, named KnetMiner, for mining genome-scale knowledge networks. Publicly accessible prototypes can be used to search the existing crop knowledge networks (KnetMiner[Internet]).

Heterogenous data sources can contain explicit references to each other, thereby making it possible to interconnect the data. In situations, where direct references do not exist between the data sources, other approaches need to be exploited for connecting the different datasets. The Ondex data integration framework provides basic text-mining capabilities to facilitate the linking of datasets lacking direct references. It analyses the unstructured PubMed abstracts of *Publication* concepts in a GSKN to establish connections between different biological entities. The text-mining based relations can create novel relationships between concepts that are not yet present in structured databases (Hassani-Pak et al., 2010).

The data integration approach is pragmatic and does not attempt to integrate every dataset that is in the public domain or exhaustively model the complex semantics of every database. Instead, our methodology focuses on integrating datasets that add value to a particular application case (e.g. candidate gene discovery) at an appropriate level of semantic complexity. Selected data sources usually contain fundamental relationships that upon integration and consecutive analysis, can yield chains of functional associations among more distant concepts. Our integration approach does not attempt to utilise raw data, but instead, it integrates processed data. For example, data from large scale genetic and transcriptomic experiments are integrated as processed and interpreted data in the form of SNP location or gene expression information; not as raw NGS data. Other tools such as Galaxy and R libraries provide the specialist tools for this stage of data analysis. We seek to complement and build on primary data analysis by supporting interpretation and knowledge discovery tools.

When developing an Ondex data integration workflow we encourage an exploratory and incremental approach, whereby each step is evaluated by opening and investigating the Ondex network in the Ondex Visualisation Toolkit. In addition, the Ondex Integrator is missing a fully-configurable Ondex parser for custom tabular data types. Before tabular files can be included in an automated Ondex integration workflow, they must first be parsed into Ondex using the Ondex Scripting Console. The process of building knowledge networks has been partially automated to include data download and data integration of RefNet using the Ondex-CLI. Using these automated steps, new versions of the knowledge networks are created on a regular basis. This approach of rebuilding knowledge networks from scratch, instead of updating the parts that have changed, has the advantage of avoiding the accumulation of legacy and obsolete data. In the future, we intend to develop methodologies to determine which parts of the knowledge network have changed after an update and which genes have new links or updated annotations of interest. This would allow for the development of automated services to inform users when new information about their genes of interest becomes available. In addition, we are considering how to exploit Linked Open Data initiatives and Semantic Web technologies which might be able to facilitate the generation of GSKNs.

The exemplars we have used demonstrate the process of building a knowledge network for a species with a sequenced genome. In many cases, especially for non-model organisms, whole genome sequences or gene models are not available. For these species, a transcriptome assembly (for example from RNA-seq data) can be used to build the knowledge network. However, a knowledge network based on a transcriptome assembly lacks genomic coordinates, and entity identifiers that are standardised across public databases. Data integration workflows and methods are therefore far more challenging for transcriptomes and the approach needs to be customised further to accommodate these specific situations.

## 5 Conclusions

Navigating the heterogeneous data landscape is a technically challenging task for many biologists and bioinformaticians that can consume an excessive amount of time pre-processing data for integrative analysis. Therefore, knowledge discovery is often hampered by the challenges of data integration and new approaches are needed to improve the efficiency, reproducibility and objectivity of these processes. We have developed transferable and readily reproducible Ondex data integration workflows to build crop knowledge networks. The process is pragmatic in that it allows a network of appropriate complexity to be developed and updated without an excessive technical and semantic burden to the user. Feasibility studies have shown that biological knowledge networks provide a suitable data structure for the effective gene and biological knowledge discovery. We have demonstrated that useful biological insights can be gained from the crop GSKNs, such as discovery of an inferred relationship between barley gene MLOC_10687.2 and a seed size phenotype. Future work will need to focus on the development of user-friendly tools, e.g. KnetMiner, that exploit such networks systematically in order to help scientists and breeders improve gene discovery for key agronomic traits such as yield, drought tolerance and disease resistance.

## Competing interests

The authors declare that they have no competing interests.

## Authors' contributions

KHP and CR conceived the project. KHP designed the GSKN workflows. MC, ME helped to refine and test the workflows. JT, MH, AL, KHP developed the Ondex components used in this work. KHP wrote the manuscript. All authors have read, reviewed and approved the manuscript for publication.

## Figures and Tables

**Table 1:** Summary of the data sources and Ondex parsers that were used to create the crop and reference knowledge networks.

Knowledge type	Data source	Data type	Ondex parser	Concept class	Relation type
Genes proteins	Ensembl Phytozome TAIR	GFF3 FASTA	FASTA-GFF3	Gene Protein	encodes
SNP	Ensembl	Tabular	Console	Gene SNP	in_proximity
GWAS	Ensembl T3 Toolbox	Tabular	Console	SNP Trait	associated_with
QTL	Gramene T3 Toolbox	Tabular	Console	QTL Trait	control
Homology	Ensembl OMA Inparanoid Blast	TAB OrthoXML	Console OrthoXML	Protein Protein	ortholog paralog has_similar_sequence
Interaction	TAIR BioGrid	Tabular PSI25	Console PSI-MI v2.5	Gene Gene	interacts_with
GO annotations	Gene Ontology GOA@EBI	GAF2.0 UniProt XML	GAF UniProt	Gene/Protein BioProc MolFunc CelComp	participates_in has_function located_in
Phenotype	TAIR UniProt NCBI	Tabular UniProt XML	Console UniProt GeneRIF	Gene/Protein Phenotype	has_observed_phenotype
Pathway	AraCyc KEGG	BioPax KEGG	BioCyc BioPAX KEGG v53	Protein Protein Complex Enzyme Compound Reaction Transport Pathway Publication	is_a part_of catalysed_by consumed_by produced_by activated_by inhibited_by published_in
Protein domain	Ensembl InterProScan HMMer	Tabular	Console	Protein Protein Domain	has_domain
Literature	PubMed	Medline XML	Medline/PubMed	Publication	NA
Literature citations	TAIR GeneRIF UniProt	Tabular UniProt XML	Console GeneRIF UniProt	Gene Publication	published_in
Gene ontology	Gene Ontology	OBO	GenericOBO	BioProc MolFunc CelComp	is_a part_of regulates
Trait ontology	Gramene	OBO	GenericOBO	Trait Ontology	is_a part_of

**Table 2:** Total number of concepts per *Concept Class* included in BarleyNet and WheatNet (Release June 2016). Note that these networks include the same RefNet.

Concept class	BarleyNet	WheatNet
Biological process	27,486	27,525
Cellular component	3787	3787
Compound	5457	2980
EC	1789	1754
Enzyme	26,698	15,150
Gene	112,091	130,815
Molecular function	9866	9919
Pathway	676	587
Phenotype	6489	6489
Protein complex	192	187
Protein domain	7032	9417
Protein	136,735	177,378
Publication	61,329	61,305
QTL	285	0
Reaction	5612	3097
RNA	1296	1296
SNP	16,030	0
TO	1314	1314
Quantitative trait	30	0
Transport	96	54
**Total**	**424,487**	**453,246**
